# An improved metrics for osteoclast multinucleation

**DOI:** 10.1038/s41598-018-20031-x

**Published:** 2018-01-29

**Authors:** Santosh K. Verma, Leonid V. Chernomordik, Kamran Melikov

**Affiliations:** Section on Membrane Biology, Eunice Kennedy Shriver National Institute of Child Health and Human Development, National Institutes of Health, Bethesda, MD 20892 USA

## Abstract

Cell-cell fusion is a key stage in development and maintenance of multinucleated cells that resorb bones and form our skeletal muscles and placenta. Here, we focus on osteoclast formation to suggest new ways of unbiased presentation of cell fusion at given conditions that combine empirical cumulative distribution function for the sizes of multinucleated cells with the total number of cell-cell fusion events, which generate these cells.

## Introduction

Multinucleated cells play an important role in human biology^1,2^. Skeletal muscles are composed of multinucleated myofibers, syncytiotrophoblast forms the outer layer of the placenta and multinucleated osteoclasts play an important role in bone remodeling and homeostasis. All these different syncytia are formed by cell-cell fusion preceded by tightly regulated differentiation processes that prime the cells for fusion^1,2^. For instance, in the case of osteoclasts both insufficient and excessive fusion may lead to serious pathological conditions such as osteoporosis, osteopetrosis and Paget’s disease^3^. Formation and growth of syncytia involves different types of fusion events – fusion between two mononuclear cells, fusion of a multinucleated cell with a mononuclear cell and fusion between two multinucleated cells^4^. For a given cell type and at a given stage in development, syncytia vary in sizes (number of nuclei per cell) and syncytia of different sizes may have different functional characteristics. Assays that characterize the efficiency of fusion and the sizes of the syncytia are routinely used in many laboratories to evaluate the efficiency of the entire differentiation processes that culminate in formation multinucleated cells^5–7^.

**Figure 1:**
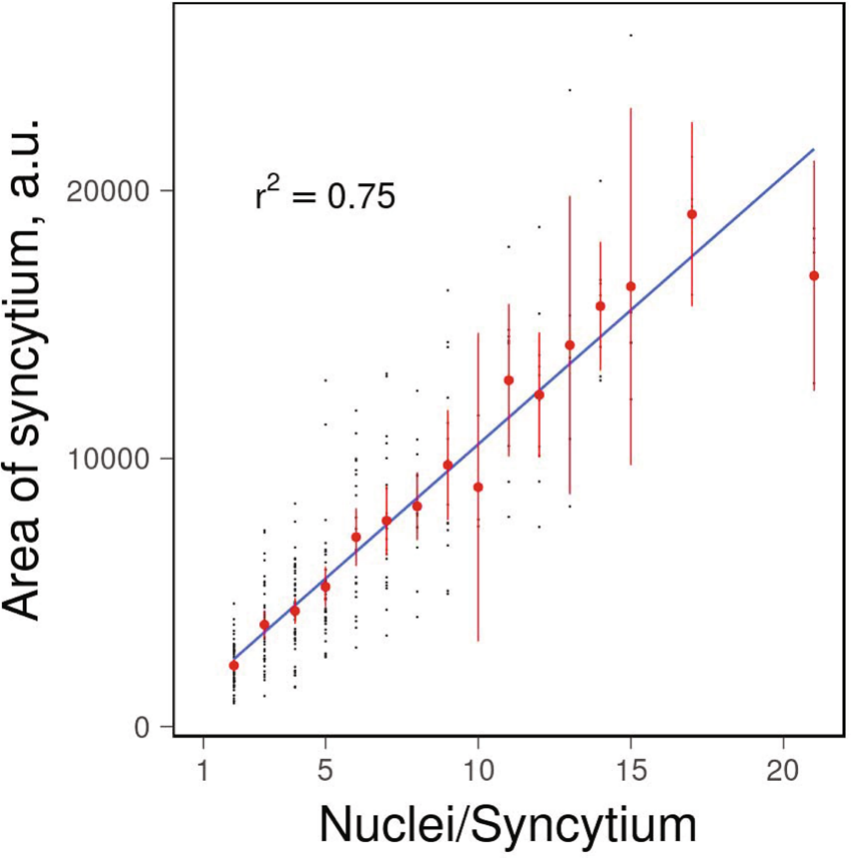
The number of nuclei in an osteoclast is proportional to its cross-sectional area. Human monocytes were induced to form osteoclasts and their cross-sectional area and number of nuclei per cell were analyzed as described in methods. Black points represent individual syncytia; red points and whiskers show mean cross-sectional area and standard error of mean for syncytia with a corresponding number of nuclei; blue line show linear fit to the data.

Formation of multinucleated osteoclasts in bone sections and in different *in vitro* models such as human monocytes or mouse bone marrow cells or RAW264.7 macrophage like cells committed to osteoclastogenesis in presence of macrophage colony-stimulating factor (MCSF) and receptor activator for nuclear factor κB ligand (RANKL) is routinely evaluated by light microscopy approaches^4–8^. Significant heterogeneity of the sizes of multinucleated cells presents a challenge in quantification of cell-cell fusion with different groups adopting various measures of osteoclast formation efficiency. Some authors report the total number of nuclei in all observed syncytia^8^, while others report average number of nuclei per syncytia together with the number of syncytia^4,9^. Either measure could be normalized to the imaged area or to the total number of nuclei (including mononuclear cells) within the field of view^8,9^. Syncytia sizes can also be assessed by measuring the surface area under each syncytium, which correlates with the number of nuclei within this cell (Fig. 1).

**Figure 2:**
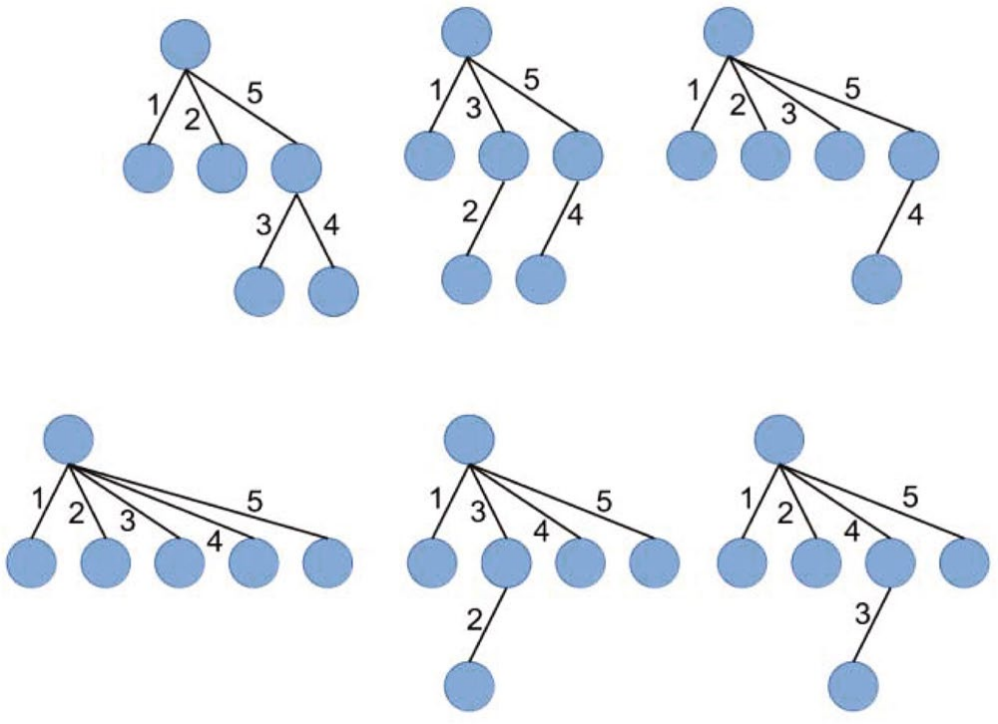
Tree representations for different pathways of formation of 6-nucleated syncytia. Blue circles represent mononuclear cells and correspond to vertices of the graph, while edges correspond to cell-to-cell fusions. Numbers show a place of a given fusion event in the sequence of fusion events. For example, in a sequence on the left of the bottom row, a syncytium is growing by sequential fusions of mononucleated cell forming 2, 3, 4, 5 and finally 6 nucleated cell. On the other hand, a sequence on the left of the top row, first, we have formation of two 3-nucleated cells and then these two cells fuse together to give six-nucleated cell. In both cases, there are 5 fusion events in total.

Here we propose a simple measure, which we will call fusion number index (FNI), to reliably quantify efficiency of cell-cell fusion in the population of heterogeneous syncytia. It is based on the fact that the number of fusion events required to form multinucleated cell with ***n*** nuclei is exactly ***n − 1***, independent of the sequence of fusion events. Indeed, formation of the cells with 1, 2 and 3-nuclei requires 0, 1 and 2 fusion events, respectively, providing a base case for the proof by induction. Syncytium with ***m*** nuclei is always formed by fusion of a cell with ***n*** nuclei and a cell with ***m − n*** nuclei, where ***0 < n < m***. Using strong inductive hypothesis that our formula is true for all positive ***n < m*** we calculate that the number of fusion events to form an ***m***-nuclear cell is ***(n − 1)*** + ***(m − n − 1)*** + ***1*** = ***m − 1***, which completes our proof. An alternative proof that formation of a multinucleated cell with ***n*** nuclei always requires ***n − 1*** fusion events comes from the graph theory. We can represent fusions as edges between vertices of a graph, where vertices correspond to mononuclear cells. Then any ***n***-nuclear cell corresponds to a connected acyclic graph with ***n*** vertices, a tree that has an ***n − 1*** edges. Different graphs represent alternative sequences of fusion events and are acyclic because cell cannot fuse with itself. For illustration, we represent all trees corresponding to formation of a 6-nucleated cell (Fig. 2).

**Figure 3:**
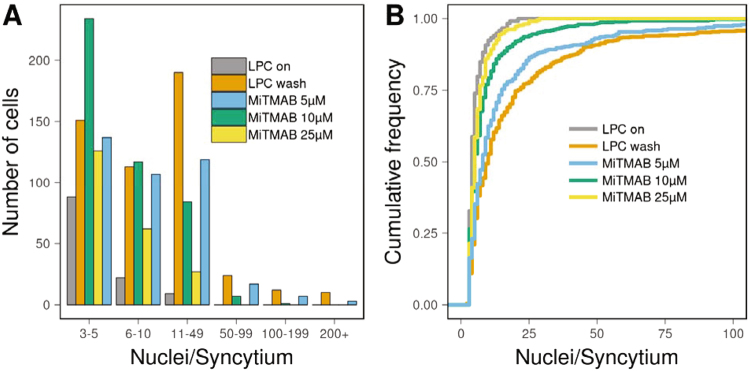
Different representations of syncytia size distribution. RAW264.7 cells were committed to osteoclastogenesis by RANKL application at t = 0. LPC was applied at t = 72 h and removed at t = 88 h (LPC wash). Number of nuclei per syncytia was analyzed at t = 89.5 h (i.e., 90 min after LPC removal) as described in methods. At the time of LPC removal cells were treated with different concentrations of MiTMAB or not treated with MiTMAB (“LPC Wash”) cells. In the negative control experiment (“LPC on”), LPC was not removed. (**A**) Bar heights represent numbers of syncytia within ranges of nuclei per syncytia denoted on X-axis. Different experimental conditions are shown in different color. (**B**) Empirical CDFs of number of nuclei per syncytia are shown in different color for different experimental conditions.

FNI is calculated as *sum(****n***_*i*_* − 1)* = *sum(****n***_*i*_*) − ****N***_*syn*_, where ***n***_*i*_ is the number of nuclei in individual syncytia, and ***N***_*syn*_ is the total number of syncytia and can be normalized to the imaged area or to the total number of nuclei (including mononuclear cells) within the field. FNI does not take into account formation of multinucleated cells by aborted division or rare cases when fused cells undergo division or fission^10^. FNI has major advantages over previously used measures for the efficiency of multinucleated osteoclasts formation. In the case of osteoclasts, fusions between two multinucleated cells may account for up to 40% of all fusion events^4^, but such fusion events do not change total number of nuclei across all syncytia and have opposite effects on average syncytia size (an increase) and on syncytia number (a decrease). In contrast, the FNI treats all types of cell fusion events, including fusions between multinuclear cells, equally. In another example, often-used approach of measuring the total number of nuclei in syncytia significantly over-represents fusions between two mononuclear cells relative to other fusion events. Let us look on a hypothetical example of a drug treatment that results in a formation of 25 bi-nuclear syncytia instead of ten five-nuclear syncytia. While there would be no change in the number of nuclei in syncytia, FNI reveals 37.5% decrease in the number of fusion events and, thus, a major inhibition of formation of multinucleated osteoclasts. In this case, again the average sizes of syncytia and their numbers change in opposite directions complicating interpretation using conventional ways of fusion representation. While FNI presents a simple-to-interpret and robust measure of the cell fusion efficiency, a detailed analysis of syncytia sizes is often desirable. This is especially true for osteoclasts, since their bone resorbing potential correlates with the cell size^11^. Histogram of the number of nuclei per cell appears to be a usual choice here^12,13^. However, a limited sample size and relatively long tailed distributions observed in real life samples lead to a wide spread use of histograms with very few bins of different size. In addition to arbitrary choice of bin limits, such histograms are difficult to interpret as the number of experimental conditions grows. As an example, in Fig. 3, we present data on inhibition of osteoclast fusion with cell permeable dynamin GTPase inhibitor MiTMAB. In this experiment, we used fusion synchronization approach developed in our earlier studies^8,14^. Ready-to-fuse RAW264.7 cells were accumulated in the presence of a reversible inhibitor of fusion LPC and fused only after LPC removal. In Fig. 3, data from the negative control, in which fusion inhibitor LPC was not removed, are shown as “LPC on” (FNI = 513). “LPC wash” represents osteoclast fusion observed upon LPC removal without MiTMAB application (FNI = 11280). As we have previously reported^8^, MiTMAB inhibits osteoclast fusion in a dose-dependent manner (FNI of 6649, 3533 and 1179 for 5 µM, 10 µM and 25 µM of drug respectively). We can observe in Fig. 3A that it is difficult to notice a consistent pattern of changes in the distribution of syncytia sizes using histogram. As a better alternative, we propose to represent distribution of syncytia sizes as empirical cumulative distribution function (CDF), which shows fraction of syncytia with number of nuclei less or equal than X (Fig. 3B). Construction of CDF does not rely on a choice of a bin size and offers a number of other advantages. In particular CDF allows an easy readout of important distributional parameters, such as median and quartiles. Moreover, such representation facilitates qualitative comparison of different distributions (Fig. 3A vs 3B). As fraction of small syncytia increases, the curves shift towards Y axis and we can easily observe trend toward smaller syncytia sizes with increasing concentrations of MitMAB.

In conclusion, the suggested measures of osteoclast fusion: FNI and CDF, are based on the same raw data of microscopy-based analysis as the currently used quantification approaches. FNI and CDF provide robust and easy to interpret readings for the cell fusion aspects of osteoclastogenesis and independently characterize both the numbers of the fusion events and the sizes of multinucleated osteoclasts *in vitro* and *in vivo*. A combination of FNI and CDF presents a full description of formation of multinucleated osteoclasts for any given conditions. While in this work we focus on the application of our metric to fusion of osteoclast precursor cells, both FNI and CDF can be used to characterize cell fusion aspects of myogenesis and syncytiotrophoblast formation.

## Methods

### Reagents

Media, FBS and antibiotics were from Life Technologies, USA. M-CSF and RANKL were procured from Cell Sciences, USA. 1-lauroyl-2-hydroxy-sn-glyecero-3-phosphocholine (LPC) was purchased from Avanti Polar Lipids, USA. Dynamin GTPase inhibitor myristyl trimethyl ammonium bromide (MiTMAB) targeting pleckstrin homology domain was purchased from Sigma, USA.

### Cell culture

Elutriated human monocytes from healthy donor who had consented to participate in the NIH IRB-approved Research Donor Program in Bethesda, MD were obtained from Department of Transfusion Medicine, National Institutes of Health, USA; all samples were anonymized. Cells were seeded on 35 mm cell culture dish and cultured in alpha-MEM supplemented with 10% (v/v) Fetal Bovine Serum (FBS) and penicillin/streptomycin. For the first 6 days monocytes were cultured in the medium with 25 ng/ml human M-CSF. After that cells were committed to osteoclastogenesis by incubating in the medium supplemented with 25ng/ml human M-CSF and 30 ng/ml human RANKL. Mouse macrophage cell line RAW 264.7 (ATCC, USA, passage number <5) were cultured in DMEM supplemented with 10% (v/v) FBS and penicillin/streptomycin. Cells were committed to osteoclastogenesis by supplementing the medium with 50 ng/ml of mouse RANKL. Both human monocytes and RAW 264.7 cells were maintained in an incubator with 5% CO_2_ in the air. Medium was refreshed every 3rd day.

### Osteoclast fusion

Osteoclast fusion assay was performed as described earlier^8^. In brief, to synchronize osteoclast fusion, LPC (170 µM for RAW 264.7 cells and 350 µM for human monocytes) was applied to fusion-committed cells at 72 hr post RANKL application. After 16 h incubation LPC was removed by five washes with LPC-free culture medium. Macrophage fusion was evaluated at 90 min after the wash. Cells were fixed with 10% (w/v) formalin solution (Electron Microscopy Sciences) and cell nuclei were labeled with Hoechst (Molecular Probes). Images were captured at room temperature on Axiovert 135 microscope (Carl Zeiss) equipped with 10×/0.30 Plan-NEOFLUAR objective lens (Carl Zeiss) and Coolsnap fx CCD camera (Photometrics) using µManager 1.4. For given condition, images of several random fields of view were captured. Osteoclast fusion was scored by counting nuclei in syncytia (>3 nuclei for RAW cells and >2 nuclei for human monocytes) and syncytia area (for human monocyte-derived osteoclasts) using semi-automated ImageJ (NIH) macro script developed in house. MiTMAB was applied to fusion committed RAW 264.7 cells at the time of LPC removal.
